# Aftereffect of single transcranial direct and alternating current stimulation on spontaneous home-cage and open-field EEG activities in a mouse model of Alzheimer’s disease

**DOI:** 10.3389/fnagi.2024.1492838

**Published:** 2024-12-16

**Authors:** Huaying Sun, Yumei Wang, Dong Yuan, Mengsi Duan, Zhuangfei Chen, Yu Fu

**Affiliations:** ^1^Medical School, Kunming University of Science and Technology, Kunming, China; ^2^School of Chinese Materia Medica and Key Laboratory of Yunnan Provincial Department of Education for Processing Research on Characteristic Prepared Drug in Pieces, Yunnan University of Chinese Medicine, Kunming, China

**Keywords:** Alzheimer’s disease, transcranial direct current stimulation, transcranial alternating current stimulation, electroencephalography, hippocampus, prefrontal cortex, mice

## Abstract

**Background:**

As a non drug and non invasive therapy, both transcranial alternating current stimulation (tACS) and transcranial direct current stimulation (tDCS) may modulate cortical rhythms and serve as potentially effective approaches to cognitive decline in Alzheimer’s disease (AD). However, studies using animal models of AD are quite limited.

**Methods:**

This study investigates the aftereffects of tACS and tDCS on brain EEG activity and associated exploratory behavior in normal aged and APP/PS1 transgenic mice (15 months old). Anodal tDCS and 10 Hz tACS (350 μA, 20 min) were applied once and EEGs were recorded from the hippocampus (Hip) and prefrontal cortex (PFC) during spontaneous home-cage state and open-field exploration.

**Results:**

A key finding was that tDCS induced significant alpha (8–12 Hz) EEG changes while tACS induced peak frequency changes in the group difference between normal aged and AD mice. However, both groups showed similar increases in theta (4–8 Hz) EEG activity during open-field exploration and increases in gamma (20–100 Hz) EEG activity in spontaneous state, suggesting that the ongoing physiological state may be related to some of the EEG changes.

**Conclusion:**

This study provides insight into the short-term aftereffects of transcranial current stimulation in the aging and AD brain and is the first animal study to compare brain activity between tACS and tDCS treatments.

## Introduction

Alzheimer’s disease (AD) is a major public health problem. It is the leading cause of dementia in the elderly. As the world’s population ages rapidly, AD is affecting people as they get older. To date, effective treatments to slow or cure the disease have not been extensively researched. Many clinical trials and drug developments for AD have produced disappointing or unconvincing results (Abbott and Dolgin, 2016; Anand et al., 2014), suggesting that new approaches, such as non-drug and non-invasive therapies, are needed to treat AD.

Transcranial current stimulation (tCS), a non-drug and non-invasive therapy, has emerged as one of the most effective methods for treating cognitive decline and modulating cortical rhythms (Liu et al., 2018; Ling et al., 2016). There are two popular tCS technologies, namely transcranial alternating current stimulation (tACS) and transcranial direct current stimulation (tDCS). Accumulating evidence suggests that both types of stimulation affect the aging brain (Hsu et al., 2015; Prehn and Floel, 2015; Abd Hamid et al., 2015). For example, theta-band frequency tACS applied to the left prefrontal cortex and left temporal cortex improved neural synchronization and working memory in older adults (Reinhart and Nguyen, 2019). In addition, a meta-analysis showed that tDCS may improve cognitive performance in older adults (Summers et al., 2016).

Aging is the major risk factor for AD, but the efficacy of tACS and tDCS in AD remains uncertain (Chang et al., 2018). Most studies to date have been conducted in clinical patients. Both types of stimulation may have potential as novel treatments for AD, but several factors contribute to mixed results in human studies, such as patient selection and sample size (Chang et al., 2018). In contrast, animal studies allow for controlled and rapid application of tCS designs and clinical settings, providing insight into the cellular mechanisms of stimulation. However, research on tACS and tDCS, similar to other translational medical studies, is very limited in animal models of AD (Nardone et al., 2015).

In this study, we report the effects of tACS and tDCS on brain EEG activity in home cages and during exploratory activity in a mouse model of AD. EEG activity was assessed in animals in spontaneous state and during open-field (OPF) performance. This is the first study to compare brain activity after tACS and tDCS in animals with AD.

## Methods

### Animals

All experimental and animal care procedures were conducted in accordance with the National Guidelines for the Care and Use of Animals and approved by the National Animal Research Authority. In addition, all experiments were approved by the local Animal Care and Use Committee.

Amyloid precursor protein/presenilin-1 double transgenic male mice (strain name: B6/JNju-Tg (APPswe, PSEN1dE9)/Nju; hereafter referred to as AD mice) and their wide-type (WT) littermates were obtained from the Nanjing Biomedical Research Institute of Nanjing University (license number SCXK [Su] 2015–0001). The transgenic mice express a chimeric mouse/human amyloid precursor protein (Mo/HuAPP695swe) and a mutant human presenilin 1 (PS1-dE9) targeted to central nervous system (CNS) neurons. The mice develop beta-amyloid (Aβ) deposits in the brain by 6 to 7 months of age. The mice used in this study were 15 months old. Genotypes were confirmed by polymerase chain reaction (PCR) genotyping at the Nanjing Biomedical Research Institute of Nanjing University using genomic DNA extracted from tail tissue samples.

Mice were individually marked with metal ear tags. During the experimental sessions, the mice were housed in groups of 1–3 animals in plastic cages (30 × 18 × 14 cm) under constant temperature (23 ± 1°C) and stable humidity with a natural light–dark cycle and free access to food and water.

Based on genotype and current stimulus, all mice were randomly divided into 4 groups: (1) WT-tDCS group, 10 mice; (2) AD-tDCS group, 9 mice; (3) WT-tACS group, 8 mice; and (4) AD-tACS group, 12 mice. A total of 39 mice were used in this study. The difference in the number of animals between groups was due to some mice with no EEG data collected for the recording areas.

### Experimental design

**Figure 1:**
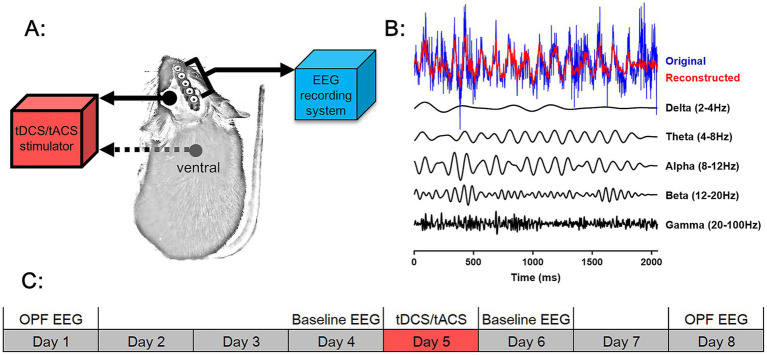
Experimental design. **(A)** Schematic of tDCS and tACS and EEG recording system on an animal. A stimulation tube was placed on the head of the animal as the active electrode for tDCS and tACS, and the counter electrode was placed over the ventral thorax of the animal. A rectangular five-pin array was connected to an EEG recording system for EEG signal acquisition. **(B)** The original EEG wave (blue), five frequency EEG waves filtered from the original EEG wave (black), and the reconstructed EEG wave from a superposition of the five frequency-filtered waves were shown. **(C)** Scheme of the study design. Open-field test EEG (OPF EEG) and baseline EEG were recorded before and after tDCS and tACS. The time course of each EEG recording and of tDCS and tACS was shown.

For the current stimulus, we used a montage similar to that described previously (Cambiaghi et al., 2010). Mice were implanted with an epicranial plastic tube for filling with saline as an active electrode prior to stimulation (Figure 1A). Meanwhile, they were implanted with metal electrodes for EEG recording (Figure 1B). After electrode implantation surgery, mice were allowed at least 1 week to recover.

The experimental routine of EEG recording and current stimulation was illustrated in the Figure 1C. Baseline EEG activities were recorded 1 day before and after stimulation, and OPF EEG activities were recorded 3–4 days before and after stimulation. On each day, the specific experimental times for EEG signal acquisition or tCS were performed in a randomized order while maintaining balance between groups. There was a 7-day interval between the two OPF experiments to avoid habituation of the mice to the OPF paradigm. In addition, all mice were allowed 3 days of habituation to the recording and stimulation procedures before the start of the routine, during which no EEG signal was recorded and no stimulation was applied.

### Surgery

Surgery was performed under pentobarbital anesthesia (60 mg/kg, i.p.; dissolved in 0.9% sodium, 10 mg/mL, Merck, Darmstadt, Germany). After a midline scalp incision, the scalp and underlying tissues were removed. First, an epicranial plastic tube (inner diameter: 4.96 mm) was implanted with its center positioned over the left Hip (AP: −1.8 mm, ML: −1.6 mm). The tube was secured to the skull with modified acrylic adhesive. Second, five burr holes were drilled into the skull. Two twisted pairs of perfluoroalkoxy (PFA)-coated stainless steel wires (diameter 0.002″, A-M Systems, WA, USA) were implanted into the brain through the two holes to serve as EEG recording electrodes to record the right Hip (AP: −1.8 mm, ML: +1.6 mm, DV: −1.7 mm from dura) and the right PFC (AP: +2.95 mm, ML: +1.5 mm, DV: −0.75 mm from dura), respectively. A stainless steel watch screw (M1.0 × L2.0 mm, RWD) was placed in contact with the dura through the hole and served as the left Ctx (control) recording electrode (AP: −3.8 mm, ML: −2.5 mm). Through the other two holes, two stainless steel watch screws were also placed in contact with the dura over the left olfactory bulb and central cerebellum, serving as reference and ground electrodes, respectively. All electrodes were attached to male pins secured in a rectangular five-by-one pin array. Finally, all electrodes and the epicranial plastic tube were secured with dental acrylic. Recording electrode locations were confirmed histologically after the EEG experiment. Data from mice with misplaced recording sites were excluded.

### Transcranial current stimulation

Prior to stimulation, mice were restrained on a homemade fixed table in the awake state with the head and torso still. For tDCS, the epicranial plastic tube was filled with saline (NaCl 0.9%), which served as the active electrode. This method was chosen to avoid polarization of the active electrode by tDCS. The counter-electrode was a saline-soaked sponge (round, diameter: 9.3 mm) placed over the ventral thorax. Anodal tDCS was applied with a current intensity of 350 μA for 20 min using a constant current stimulator (Cerebooster, Droian, Hangzhou Zhuo An Zi network technology Co. Ltd). This intensity corresponds to a current density of 1.81 mA/cm^2^ (0.35 mA/0.193 cm^2^). To avoid a stimulation-break effect, the current intensity was linearized within 10 s rather than being directly switched on and off. For tACS, the mice received the stimulation in the form of a sine wave. tACS was delivered at 350 μA and at 10 Hz for 20 min. All other operations on the mice in the tACS groups were identical to those in the tDCS groups. Animals were kept awake and restrained during current stimulation to prevent interaction between the effects of current stimulation and anesthesia.

### EEG recording protocol

The EEG signal acquisition system consisted of an RHD2132 amplifier, RHD2000 USB interface board, and RHD2000 Interface GUI software (Intan Technologies, Los Angeles, CA, USA). Data were acquired at a sampling rate of 1 kHz. All electrodes on each mouse were connected by a cable to the amplifier, then to the interface board, and finally to the computer. The cable was suspended from a helium balloon to allow the mice to move freely. Recordings were made in a shielded cage (80 × 70 × 100 cm). The behavior of the mice in the cage was monitored by a CCD camera mounted on the ceiling. The video signals were then displayed and stored using video capture software.

For baseline EEG recording, a new home cage was placed inside the shielding cage and the floor of the home cage was covered with sawdust. This condition is similar to their daily living conditions. EEG signals were recorded from the mice in spontaneous state for 30 min during their waking period.

For OPF EEG recording, an open-field apparatus (40 × 40 × 30 cm) was placed inside the shielding cage. The apparatus was made of PVC board with black walls and white floor. During the experiment, the mice were gently placed in the apparatus at each corner and allowed to move freely in the apparatus for 5 min. Meanwhile, EEG signals were collected for each mouse. After the experiment, the apparatus was cleaned with 75% alcohol to eliminate the influence of mouse feces and odor.

### Data analysis

As described previously (Fu et al., 2019), EEG signals were examined by offline analysis and spectral analysis was performed using MATLAB.

EEGs were recorded by the Intan system as rhd data files. EEG spectral analysis was performed using the mtspecgramc function from the Chronux toolbox^1^ in MATLAB. This function does not use a 50 Hz notch filter. The function calculates the multitaper spectrum over a moving window with user-adjustable time width and step size. The padding factor for the Fast Fourier Transform was set to 1 (padding to 1,024 points). The spectrum was calculated for the frequency range 0–120 Hz. The time-bandwidth product was set to 3, with 5 tapers used for estimation. The spectrum was averaged over the trials.

For frequency band power analysis, each rhd data file was first divided into segments, with each segment consisting of 1,024 sample points. The segments were filtered (with a 50 Hz notch filter) for the following EEG frequency bands: (1) delta, 2–4 Hz; (2) theta, 4–8 Hz; (3) alpha, 8–12 Hz; (4) beta, 12–20 Hz; and (5) gamma, 20–100 Hz (Figure 1B). For each frequency band, the absolute power of each segment was calculated as P = ∑*ꭕ*^2^/1,024. The relative power (RP) was calculated as the percentage of power relative to the total power of all frequency bands. The average of all segments was used for further statistical analysis (Fu et al., 2019; Yuan et al., 2024).

In the present study, two methods of EEG data analysis, power spectrum and relative power, were used to investigate the effects of tDCS and tACS on the AD brain. Both analyses are commonly used in EEG studies and in the investigation of the pathological mechanisms of AD (Fu et al., 2019; Yuan et al., 2024; Wang et al., 2020; Giustiniani et al., 2023). In addition, to study the effects of tCS on the EEG, the spectrum and power differences between before and after stimulation (i.e., post minus pre) were calculated for each animal for each recording state (baseline and OPF). These differences were compared between WT and AD groups. In addition, the frequency corresponding to the peak of the EEG spectral difference was also compared between the two groups.

Behavioral data in the OPF were expressed as: (1) total ambulation; (2) percentage of ambulation in the central area; and (3) number of rearing times. Data from (2–3) were indices of anxiety level, while (1) was an index of locomotor activity.

### Statistical analysis

The Shapiro–Wilk method was first used to test the normality of all data. Parametric and non-parametric tests were performed for normally and non-normally distributed data, respectively. Paired t-test and Wilcoxon signed-rank test were used to compare before and after data from the same group for parametric and nonparametric tests, respectively. Independent t-test and Mann–Whitney U test were used to compare data from unpaired groups for parametric and nonparametric tests, respectively. Two-way ANOVA (ANOVA-2) and Scheirer-Ray-Hare test were used to analyze main and interaction effects of two factors (e.g., group and stimulus) for parametric and nonparametric tests, respectively. In addition, repeated measures ANOVA (ANOVA-R) was used to analyze within-group (e.g., pre and post) and between-group effects for parametric tests. In the case of this analysis for nonparametric tests, the Wilcoxon signed-rank test and the Mann–Whitney U test were used for within-group and between-group effects. To examine the correlation between changes in EEG activity and changes in behavioral performance of the OPF, partial correlation analysis (Zhou et al., 2022) was used with changes in locomotion as a covariate in the WT and AD groups. For each group, all tDCS and tACS data were pooled for analysis. Data are expressed as mean ± SEM. *p* values of <0.05, <0.01, and <0.001 were considered significant, highly significant, and very highly significant, respectively.

## Results

### tACS and tDCS altered EEG power spectra in both AD and WT mice

**Figure 2:**
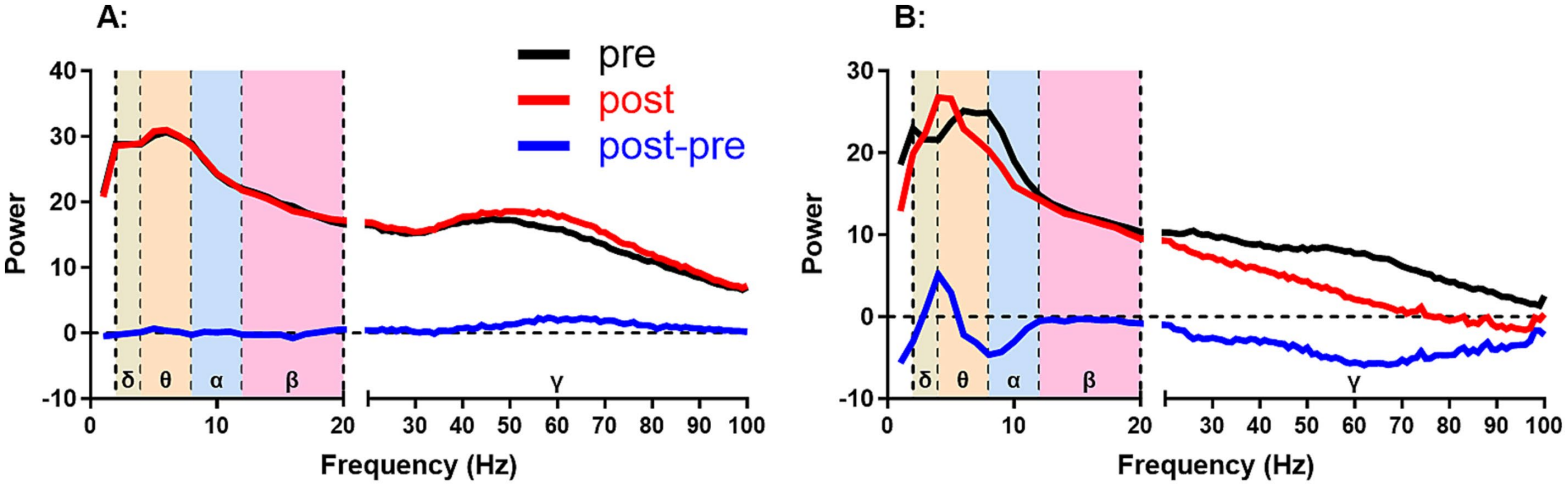
Two examples of calculating power change from power spectra. The power change was calculated from the post-stimulus power (post) minus the pre-stimulus power (pre), i.e., post-pre. **(A,B)** show smaller and larger changes after stimulation than before stimulation, respectively. Positive and negative values represent increases and decreases in power change, respectively. δ, θ, α, β, and γ represent delta (2–4 Hz), theta (4–8 Hz), alpha (8–12 Hz), beta (12–20 Hz), and gamma (20–100 Hz) frequency bands, respectively.

First, we analyzed the changes in the total power spectra of the EEG of Hip, PFC and Ctx in the home cage (spontaneous state) and during the open-field task (OPF state). The amount of power change is equal to the post-stimulation power minus the pre-stimulation power (Figure 2). A positive value represents an increase in power and a negative value represents a decrease in power.

**Figure 3:**
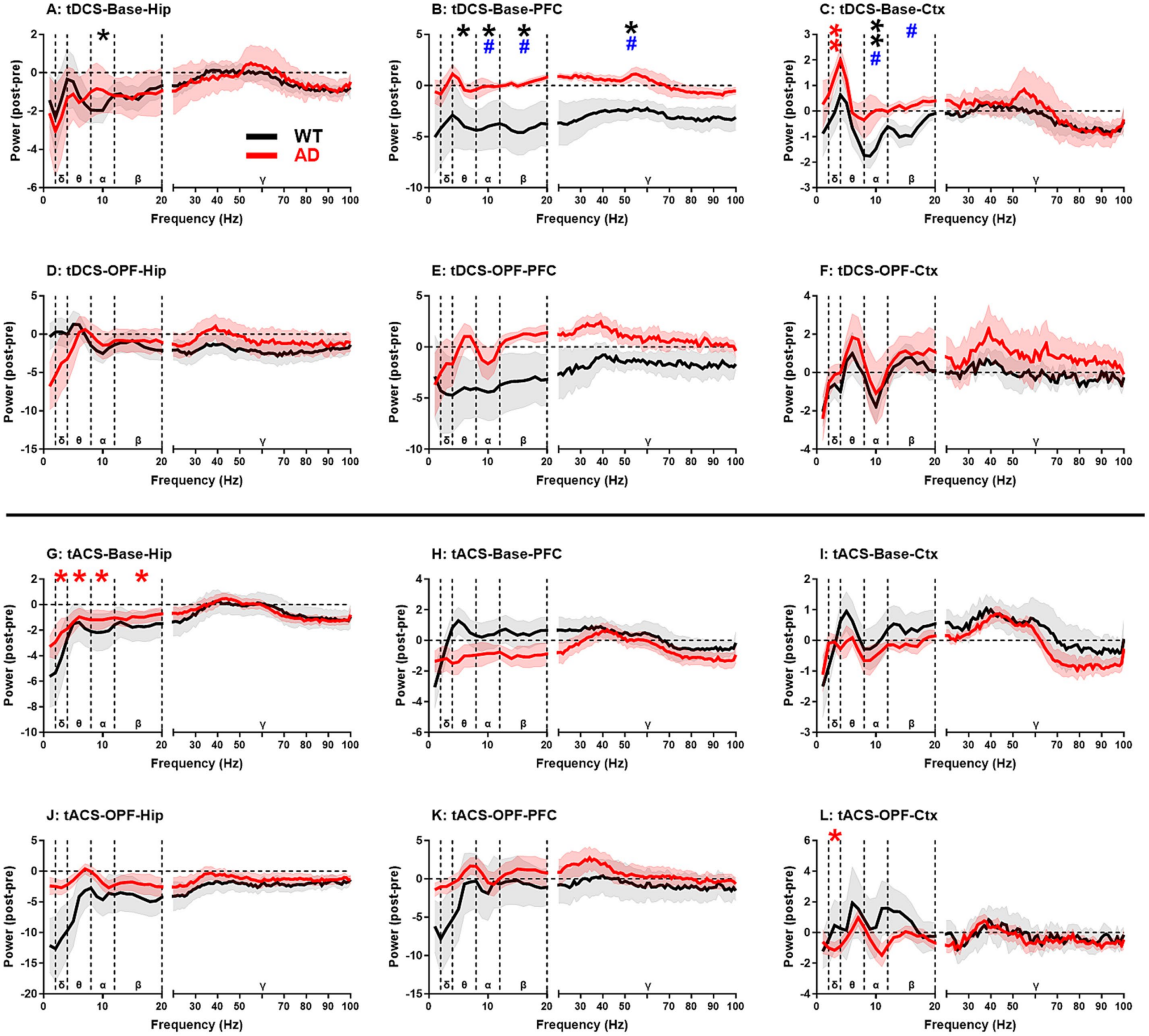
EEG power spectrum changes in hippocampus (Hip), prefrontal cortex (PFC), and general cortex (Ctx) after tDCS and tACS in WT and AD animals. **(A–C)** Baseline EEG power spectrum changes after tDCS. (D-F) Open-field test EEG power spectrum changes after tDCS. (G-I) Baseline EEG power spectrum changes after tACS. **(J–L)** Open-field test EEG power spectrum changes after tACS. δ, θ, α, β, and γ represent delta (2–4 Hz), theta (4–8 Hz), alpha (8–12 Hz), beta (12–20 Hz), and gamma (20–100 Hz) frequency bands, respectively. Mean power changes within each frequency band were used for statistical significance analysis. *p < 0.05 and ** *p* < 0.01 indicate significance of post-stimulus power compared to pre-stimulus power. Black and red stars represent the significance of WT and AD animals, respectively. #p < 0.05 for comparisons between WT and AD animals. Base: baseline EEG; OPF: open-field test EEG.

Power spectrum analysis (Figure 3) showed that, after tDCS, there were significant group differences in the alpha-gamma frequency bands in PFC (*p* < 0.05 for all; Figure 3B) and in the alpha-beta frequency bands in Ctx (p < 0.05 for both; Figure 3C) in spontaneous state. This difference can be attributed to a marked reduction in the total power spectra of the frequency bands in WT mice after stimulation. In particular, the alpha frequency band showed a significant decrease in all three brain regions (*p* < 0.05 or 0.01; see figures for detailed significance of post vs. pre comparisons and the same below; Figures 3A–C). This may indicate that WT mice were more sensitive to tDCS than AD mice. In AD mice, the most notable change was a significant increase in the delta band of Ctx after tDCS (*p* < 0.01; Figure 3C). In the OPF, no group difference was found between the two genotypes of animals, suggesting that the effects of tDCS may also be related to the physiological state of the animals.

On the other hand, after tACS, no significant group differences were found between WT and AD mice, indicating that the influence of tACS is less powerful than that of tDCS. However, AD mice seemed to be more sensitive to tACS compared to WT mice (Figures 3G–L). In spontaneous state, significant reductions in EEG activity in delta-beta frequency bands were observed in Hip of AD mice (*p* < 0.05 for all; Figure 3G), while in OPF, a significant decrease in delta band was observed in Ctx of AD mice (*p* < 0.05; Figure 3L). This may indicate that these changes are more likely due to intrinsic changes within the AD brain rather than being closely related to the activity state of AD mice.

### The frequency corresponding to peak change of EEG power altered in OPF between WT and AD mice

According to Figure 3, we found that the overall power spectrum changes differently in different EEG frequency bands. The maximum changes in the power spectra (peak change) were further analyzed between genotype groups and stimuli. However, in no recording area did the peak changes show a significant main effect of group and stimuli, nor did the interaction effect of the two factors (*p* > 0.05 for all; data not shown).

**Figure 4:**
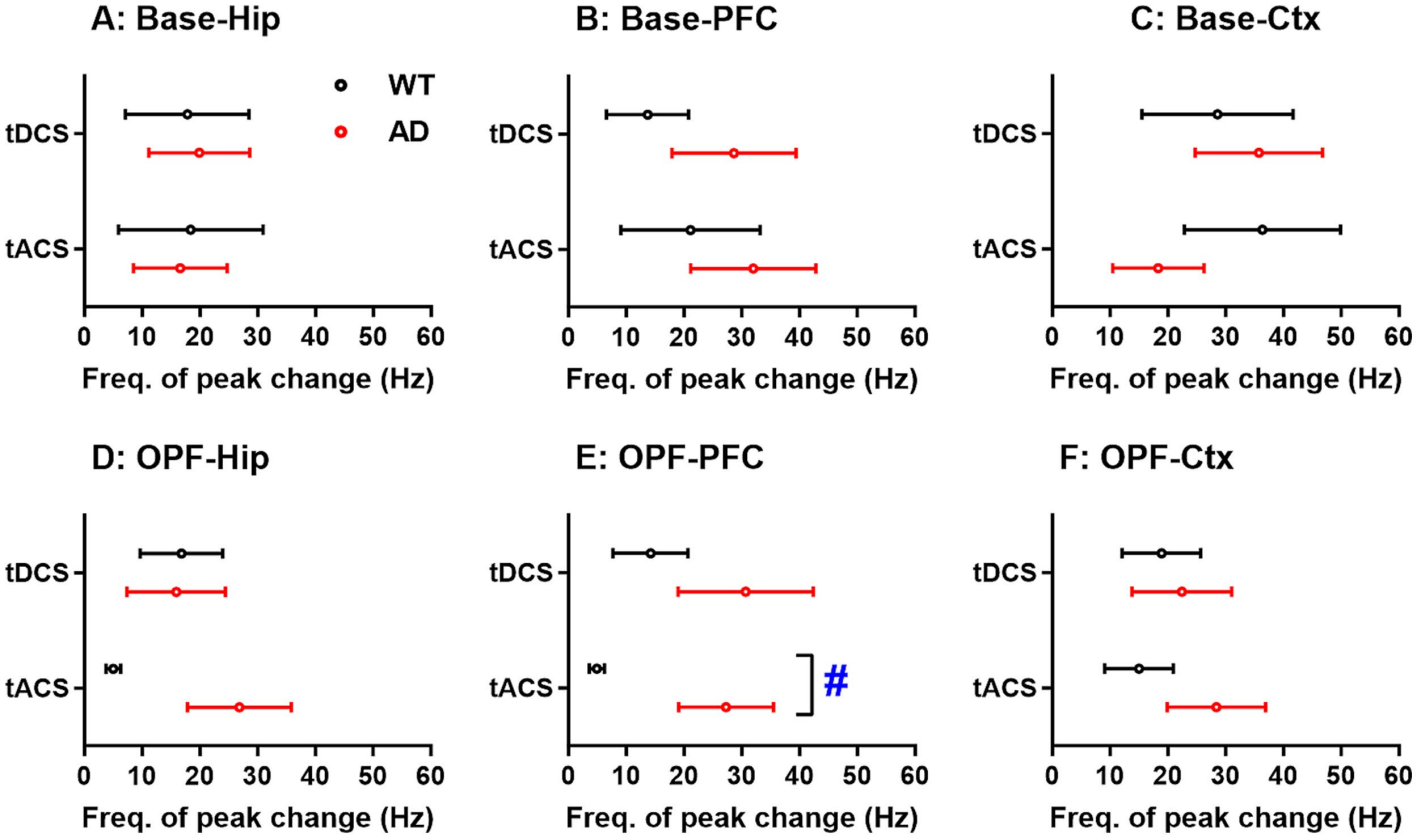
Frequency differences in peak changes of EEG power spectra in hippocampus (Hip), prefrontal cortex (PFC), and general cortex (Ctx) of WT and AD animals after tDCS and tACS. (A-C) Frequency differences in peak changes of baseline EEG power spectra. (D-F) Frequency differences in peak changes of open-field test EEG power spectra. The x-axis is the frequency corresponding to the peak change (Freq. of peak change). #p < 0.05 for comparisons between WT and AD animals. Base: baseline EEG; OPF: open-field test EEG.

The frequencies corresponding to the peak change were further analyzed for each genotype group and stimulus (Figure 4). In the open-field task, a significant main effect of group was found in PFC (*p* < 0.05; Figure 4E). The frequencies of peak changes were significantly lower in WT animals (4.87 ± 1.32 Hz) compared to the high frequencies in AD animals (27.25 ± 8.19 Hz) after tACS (*p* < 0.05). For the Hip, these frequencies were also lower in WT animals (5.00 ± 1.27 Hz) compared to the high frequencies in AD animals (26.83 ± 8.97 Hz) after tACS (Figure 4D). However, the main effects of group and stimulus and their interaction were not significant (*p* = 0.387 and 0.545 for group and stimulus, and *p* = 0.093 for group × stimulus).

### Changes in relative power of EEG activity in both AD and WT mice after tACS and tDCS stimulation

**Figure 5:**
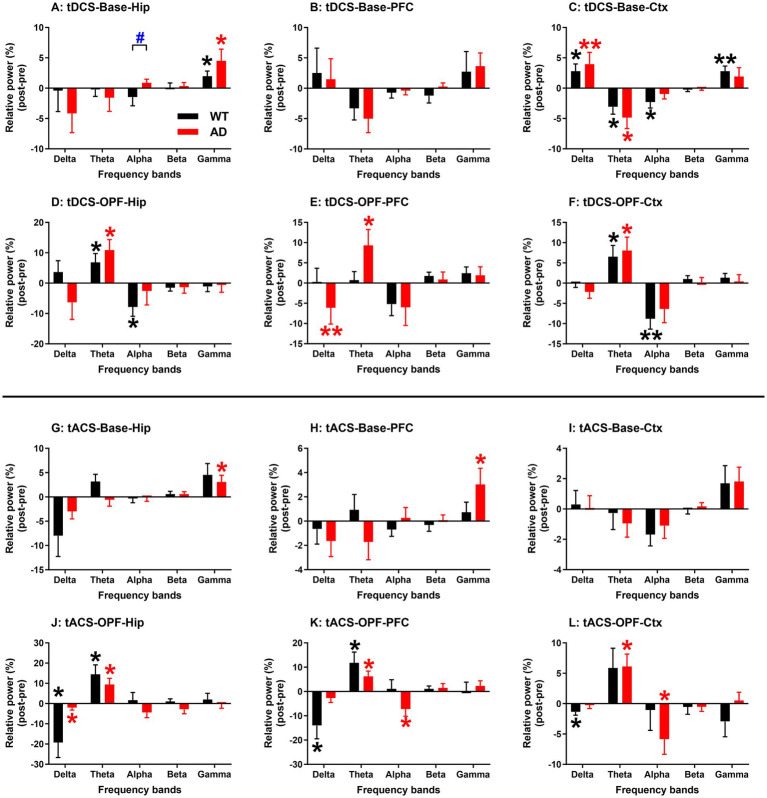
EEG relative power changes in hippocampus (Hip), prefrontal cortex (PFC), and general cortex (Ctx) after tDCS and tACS in WT and AD animals. **(A–C)** Baseline EEG relative power changes after tDCS. **(D–F)** Open-field test EEG relative power changes after tDCS. **(G–I)** Baseline EEG relative power changes after tACS. **(J–L)** Open-field test EEG relative power changes after tACS. EEG relative power changes in five frequency bands were analyzed: delta (2–4 Hz), theta (4–8 Hz), alpha (8–12 Hz), beta (12–20 Hz), and gamma (20–100 Hz). *p < 0.05 and ** p < 0.01 indicate significance of post-stimulus power compared to pre-stimulus power. Black and red stars represent significance from WT and AD animals, respectively. Base: baseline EEG; OPF: open-field test EEG.

The RPs of all five frequency bands from all three recording regions were analyzed and then compared between genotype groups for tDCS and tACS (Figure 5). In general, changes in EEG RPs after stimulation were associated with the different states of the animals. In spontaneous state, RP changes in the gamma band showed a significant increase (*p* < 0.05 or 0.01; see figures for detailed significance of post vs. pre comparisons and the same below; Figures 5A,C,G), and during OPF, RP changes in the theta band showed a significant increase (*p* < 0.05 for all; Figures 5D–F,J–L).

In addition, a significant group difference was observed for RP changes in the alpha band in Hip after tDCS in spontaneous state (*p* < 0.05; Figure 5A). However, for this frequency band, most RP changes after tDCS or tACS were negative in both WT and AD mice (Figures 5C,D,F,K,L). The reason for the group difference may then be due to the alpha increase in Hip of AD mice (Figure 5A), which was not observed in PFC and Ctx of AD mice (Figures 5B–L).

For the theta frequency band, both WT and AD mice showed increased RP after tDCS or tACS during OPF (Figures 5D–F,J), but decreased RP in spontaneous state (Figure 5C). For the delta frequency band, a similar decreasing trend was generally observed during OPF (Figures 5E,J–L). Conversely, in spontaneous state, RP changes in the delta band of Ctx increased in both WT and AD mice (Figure 5C). These results may indicate a state/task dependent EEG change within the theta and delta frequency bands. In addition, RP changes in the gamma band also showed consistent variations between WT and AD mice (Figures 5A,C,G), especially in Hip after tDCS (Figure 5A). In short, RP changes after tDCS and tACS showed more consistent characteristics in both types of mice.

Table 1 summarizes the EEG changes in five frequency bands in all three recording areas for WT and AD mice after tACS and tDCS.

**Table 1:** Comparison of absolute (spectral power) and relative EEG power changes between WT and AD mice in five EEG frequency bands.

				Hip EEG activity					PFC EEG activity					Ctx EEG activity				
				Delta	Theta	Alpha	Beta	Gamma	Delta	Theta	Alpha	Beta	Gamma	Delta	Theta	Alpha	Beta	Gamma
Spectral power (Sp)	tDCS	Base	WT			−				−	−	−	−			−−		
			AD											++				
			vs.								#	#	#			#	#	
		OPF	WT															
			AD															
	tACS	Base	WT															
			AD	−	−	−	−											
		OPF	WT															
			AD											−				
Relative power (RP)	tDCS	Base	WT					+						+	−	−		++
			AD					+						++	−			
			vs.			#												
		OPF	WT		+	−									+	−−		
			AD		+				−−	+					+			
	tACS	Base	WT															
			AD					+					+					
		OPF	WT	−	+				−	+				−				
			AD	−	+					+	−				+	−		

+*p* < 0.05 or ++*p* < 0.01, EEG activity significantly increased after tDCS or tACS. −*p* < 0.05 or −−*p* < 0.01, EEG activity significantly decreased after tDCS or tACS. #*p* < 0.05, significance observed for comparison between WT and AD animals. Delta: 2–4 Hz; Theta: 4–8 Hz; Alpha: 8–12 Hz; Beta: 12–20 Hz; Gamma: 20–100 Hz. Base: baseline EEG activity; OPF: open-field test EEG activity; WT: wild-type mice; AD: APP/PS1 mice; Hip: hippocampus; PFC: prefrontal cortex; Ctx: general cortex.

### Correlation patterns between EEG and behavior performance in OPF in AD and WT mice after tACS and tDCS stimulation

**Figure 6:**
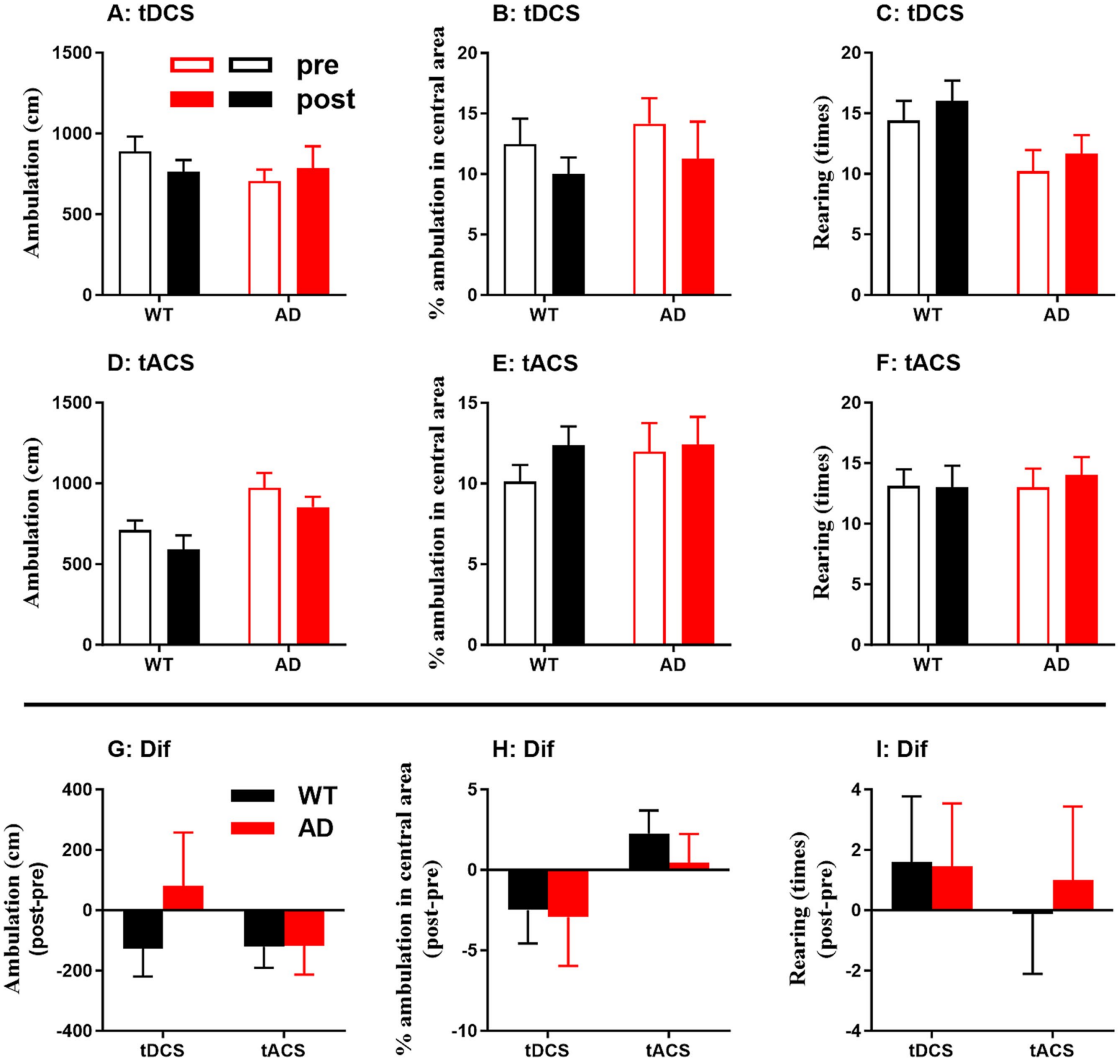
Behavioral outcome in the open-field (OPF) test in WT and AD animals. **(A–C)** Behavior before and after tDCS. **(D–F)** Behavior before and after tACS. (G-I) Behavioral changes (Dif) were calculated from post-stimulation behavioral indices (post) minus pre-stimulation behavioral indices (pre), i.e., post-pre. Total ambulation, percentage of ambulation in the OPF center, and rearing times of the animals are shown.

Behavioral performance, including total ambulation, percentage of ambulation in the central area and number of rearing times, in the OPF showed no significant changes after tDCS and tACS for each genotype group (*p* > 0.05 for all; Figure 6A–F). In addition, changes in behavioral performance showed no significant difference between genotype groups and stimuli (*p* > 0.05 for all; Figures 6G–I). These behavioral data from tDCS and tACS were then combined to determine any correlation with EEG characteristics.

After controlling for the influence of locomotor activity, partial correlation analysis between EEG activity changes (relative power) and OPF behavioral changes was performed for WT and AD mice (Table 2). Changes in the percentage of ambulation in central area were negatively correlated with Ctx EEG activity changes in the delta band in WT mice, but positively and negatively correlated with Hip EEG activity changes in the delta and alpha bands, respectively, in AD mice (see correlation coefficients and *p* values in the table; same below). In addition, changes in the number of rearing times were negatively correlated with gamma EEG activity changes in all three recording areas in WT mice, but positively correlated with Ctx EEG activity changes in the alpha band in AD mice.

**Table 2:** Partial correlation analysis controlling for locomotor changes between EEG activity changes and OPF behavioral changes in WT and AD animals.

OPF behavioral changes (post-pre)	EEG activity changes (post-pre)	Hip EEG activity				PFC EEG activity				Ctx EEG activity			
		WT (*n* = 18)		AD (*n* = 21)		WT (*n* = 18)		AD (*n* = 21)		WT (*n* = 18)		AD (*n* = 21)	
		r	*p*	r	*p*	r	*p*	r	*p*	r	*p*	r	*p*
% Ambulation in central area	Delta	−0.094	0.720	0.603	0.005**	−0.158	0.544	0.195	0.411	−0.491	0.045*	0.400	0.080
	Theta	−0.195	0.453	−0.054	0.820	0.027	0.919	0.191	0.420	−0.076	0.773	0.078	0.744
	Alpha	0.325	0.203	−0.446	0.049*	0.248	0.338	−0.371	0.107	0.402	0.110	−0.173	0.466
	Beta	0.085	0.745	−0.072	0.764	−0.133	0.610	0.137	0.565	−0.180	0.490	−0.004	0.988
	Gamma	0.076	0.773	−0.175	0.462	−0.007	0.979	−0.058	0.807	−0.187	0.473	−0.102	0.670
Rearing	Delta	0.091	0.727	−0.017	0.944	0.083	0.753	0.034	0.888	−0.317	0.216	0.200	0.397
	Theta	0.130	0.620	−0.161	0.496	0.269	0.296	−0.103	0.666	0.286	0.266	−0.199	0.401
	Alpha	0.151	0.563	0.361	0.118	0.167	0.522	0.382	0.097	0.321	0.209	0.448	0.047*
	Beta	−0.297	0.248	−0.095	0.692	−0.428	0.086	−0.144	0.544	−0.360	0.155	−0.291	0.213
	Gamma	−0.536	0.026*	−0.188	0.428	−0.580	0.015*	−0.389	0.090	−0.563	0.019*	−0.354	0.126

Post-pre: Values after tDCS or tACS minus values before tDCS or tACS. Changes in EEG activity were calculated from relative power. r: Partial correlation coefficients; *p* values (*p < 0.05 and **p < 0.01) from partial correlation analysis. Delta: 2–4 Hz; Theta: 4–8 Hz; Alpha: 8–12 Hz; Beta: 12–20 Hz; Gamma: 20–100 Hz. OPF: open-field test; WT: wild-type mice; AD: APP/PS1 mice; Hip: hippocampus; PFC: prefrontal cortex; Ctx: general cortex.

## Discussion

The present study provides a direct comparison of the electrophysiological effects of single-session tDCS and tACS between WT and AD mice. Our main findings are: (1) There was a significant difference between WT and AD mice in the spontaneous absolute alpha-gamma band power change after tDCS, and the frequency corresponding to the peak change in EEG power during OPF task was different after tACS; (2) For relative power, the EEG change in WT and AD mice showed similar increases or decreases in theta, alpha, and gamma bands after tCS under different physiological states; (3) Although exploratory behavior did not appear to change at the current stage of aging, the correlation patterns between changes in EEG activity and behavioral performance in the OPF were different, showing significant correlation in the high-frequency gamma band in WT, but low-frequency delta and alpha in AD.

The cerebral cortex generates complex oscillatory activity that is involved in a variety of higher brain functions. Among the EEG changes observed in different frequency bands, a consistent finding in both power spectrum (absolute power) and relative power is that tDCS reduces alpha activity in Hip or Ctx of normal aging mice. The mechanisms underlying the neuromodulatory effects of tDCS remain highly controversial and have been the subject of numerous studies. Several experiments suggest that neurons respond to tDCS-induced membrane polarization changes (Liebetanz et al., 2002), leading to an increase in spontaneous neuronal firing rates after anodal stimulation. Aftereffects can last for minutes or hours, depending on the intensity and duration of stimulation (Mangia et al., 2014). If anodal tDCS is associated with increased cortical excitability, a decrease in alpha amplitude would be expected (Spitoni et al., 2013). Alpha activity has been associated with approach and withdrawal motivation (Gray, 1970). Increased alpha amplitude is often associated with cortical deactivation and inhibition (Klimesch et al., 2007; Gollan et al., 2014). Specifically, less relative left frontal alpha activity (greater left activation) has been associated with increased sensitivity to behavioral activation or increased approach motivation when goal-directed action is indicated (Coan and Allen, 2003; Sutton and Davidson, 1997). While the lack of change in alpha frequency in AD mice after tDCS may be indicative of the different physiological properties in spontaneous state.

For the aftereffect of tDCS and tACS in relative power change, a notable increase in the gamma band was observed in both AD and WT mice in spontaneous state. This increase in gamma synchronization reflects increased cortical excitability (Schroeder and Lakatos, 2009). Cortical gamma activity is thought to result from synchronous activity of fast-spiking inhibitory neurons in the cortex (Cardin et al., 2009) and has been linked to the integration of temporally correlated neural events (Wang, 2010) as a prerequisite for higher-level cognitive processing and attentive wakefulness (Clayton et al., 2015). It is plausible that depolarization of cortical structures after anodal stimulation (Medeiros et al., 2012) facilitates fast spiking and gamma EEG activity. However, the increased gamma activity occurred only in AD after tACS, which may indicate a specific response of AD. tACS has been introduced as a promising tool capable of enhancing brain oscillations, with the offline effect of tACS lasting up to 70 min (Kasten et al., 2016), which may depend on synaptic plasticity. Beta-tACS was found to increase cortical excitability and beta oscillations (Wischnewski et al., 2019), but 10 Hz tACS decreased corticospinal excitability (Lafleur et al., 2020). To the best of our knowledge, there are currently no animal studies using tACS as a treatment for AD.

When the EEG changes in the theta band after tDCS and tACS were further examined, the altered patterns seemed to be more relevant to the activity state of the animal. For example, an increase in the theta band was found in both WT and AD after either tDCS or tACS, but only in the exploratory condition. The theta rhythm (4–12 Hz) represents prominent oscillations recorded in Hip and surrounding limbic structures during exploration and REM sleep (Vanderwolf, 1969; Kitchigina, 2018). Rhythmic oscillatory activity at the theta frequency (4–12 Hz) in Hippocampus has long attracted attention because of its implications for various brain functions, including spatial cognition (Shin et al., 2005). Previous EEG evidence has shown increased brain theta power and/or decreased delta power in mice during overt exercise compared to quiet wakefulness (Lopez et al., 2020). Thus, an increase in theta activity following tDCS and tACS in the open-field may indeed indicate heightened attention following stimulation during exploration.

On the other hand, the frequency of EEG peak changes in PFC of WT animals during OPF was significantly lower than that of AD animals after tACS, i.e., 4.87 Hz versus 27.25 Hz, respectively. Interestingly, in spontaneous state, it was 21.13 Hz versus 32.00 Hz for WT and AD animals, respectively. Thus, the main reason for the apparent difference in frequency during OPF may be that WT animals had a reduced frequency after tACS. In addition, a similar decrease was observed in Hip of WT animals, although the difference with AD animals was not statistically significant. It appears that tACS had a greater effect on the frequency of EEG peak changes in WT than in AD. Brain rhythms are known to be non-stationary in the sense that their peak frequency changes transiently over time. A previous study found that the maximum EEG peak frequency differed between AD and frontotemporal lobar degeneration (Goossens et al., 2017). However, to our knowledge, the frequency of peak changes after tCS has not been investigated in either normal aging or pathological aging such as AD. The present finding is consistent with one of our previous studies. A gamma-aminobutyric acid receptor B-type agonist, baclofen, induced greater EEG peak changes in Hip and general cortex in WT than in AD mice (Yuan et al., 2024). Importantly, this EEG change was observed in the delta low frequency band and the animals were of the same age. However, future studies could further test whether the peak change may indicate different sensitivity of frequency bands to tCS in WT and AD animals.

In addition, the correlation between EEG activity changes and behavioral indices in the OPF may further highlight the difference between WT and AD animals after tCS stimulation. Here, the behavioral indices in the OPF were mainly reflected in the percentage of ambulation in central area and the number of rearing times of the animals. The rearing times primarily reflect the exploratory behavior of the animals, but are also related to some extent to their anxiety level. Both indices increase as the animals’ general anxiety decreases. In psychiatric disorders including anxiety, conflicting results are often reported for each EEG frequency band (Newson and Thiagarajan, 2019). However, the prevailing finding is a tendency to increase low frequency EEG activity (delta-theta) and decrease high frequency EEG activity (alpha-gamma; Newson and Thiagarajan, 2019). In the present study, rearing times was negatively correlated with gamma EEG activity in Hip and PFC in WT animals, e.g., anxiety decrease vs. gamma increase. In addition, the percentage of ambulation in center area was positively and negatively correlated with delta and alpha EEG activity in Hip, respectively, in AD animals, e.g., anxiety decrease vs. delta decrease and alpha increase. Thus, the relationship between anxiety and EEG frequency activity in our WT and AD animals is indeed consistent with the prevailing findings in psychiatric disorders (Newson and Thiagarajan, 2019). However, the detailed reason why the correlations were reflected in different frequency bands in WT and AD animals needs further investigation. It may be related to the fact that the dominant EEG frequency was altered by the AD pathology, such as EEG slowing, which is an important feature of the AD brain (Giustiniani et al., 2023; Dauwels et al., 2010).

Several limitations must be acknowledged. First, the present study showed the lack of behavioral improvement despite EEG changes after tDCS and tACS. Similar results have been observed in previous reports. For example, tACS had little effect on scale scores and Abeta levels in AD patients, but led to an increase in gamma EEG power spectrum, an increase in cerebral blood flow, a decrease in p-Tau burden, and a decrease in microglial activation (Dhaynaut et al., 2022; Sprugnoli et al., 2021). In addition, although tDCS could decrease Abeta levels and increase neurovascular unit function, it did not show a significant effect on some behavioral measures, such as visuospatial ability and novel object recognition (Kim and Yang, 2023; Luo et al., 2020). It may be that the effects of a single stimulation session are too short-lived to produce measurable behavioral benefits, or that the behavioral task used in this study is not sensitive enough to detect subtle cognitive changes in AD animals. Future studies may optimize the experimental design, such as increasing the number of sessions of tCS and using multiple behavioral tasks, to evaluate its potential benefits on behavior. Second, the present study showed that the correlations between EEG and OPF behavior were different after tCS in WT and AD animals, which is consistent our previous report. This study showed different correlation patterns between EEG and memory performance in WT and AD animals (Zhou et al., 2022). However, to date, there are very few studies that directly correlate indices at different levels, especially correlating the electrophysiological indices with other indices such as behavioral and pathological indices. Therefore, we could not provide a very clear explanation for this correlation result. Third, in addition to EEG recordings from the Hip and PFC, the present study also recorded EEG from the general cortex (i.e., Ctx) as a control. For this recording, a screw was used to collect the cortical response over a large area of the brain. Given some significant findings in Ctx, some of which were even different from those in Hip and PFC (e.g., delta increase and theta decrease in Ctx under spontaneous state), there is a need to further investigate the role of tCS on multiple brain areas and to consider other brain areas that are less susceptible to tCS as a control cortex.

In conclusion, because the animals used in this study were middle-aged, the similar and different effects of tDCS and tACS on EEG activity in WT and AD animals may indicate their similarity and difference in normal aging and pathological aging of AD. The similarity is mainly reflected in the increased relative power in theta and gamma frequencies, suggesting a possible cognitive enhancement in both types of aging. The difference is reflected in many aspects, such as different effects of tDCS on power spectrum especially at alpha frequency, different frequencies of peak power spectrum changes of tACS, and EEG and behavioral correlations reflected at different frequencies after stimulation. However, what exactly these differences in tDCS and tACS mean between normal and AD pathological aging requires further investigation. In summary, this study provides evidence for the short-term aftereffects of tCS in the aging and AD brains.

## Data Availability

The raw data supporting the conclusions of this article are available upon request from the corresponding author.

## Footnotes

^1^ http://chronux.org/
